# Micropowder Ca_2_YMgScSi_3_O_12_:Ce Silicate Garnet as an Efficient Light Converter for White LEDs

**DOI:** 10.3390/ma15113942

**Published:** 2022-06-01

**Authors:** Anna Shakhno, Anton Markovskyi, Tetiana Zorenko, Sandra Witkiewicz-Łukaszek, Yevheniya Vlasyuk, Andres Osvet, Jack Elia, Christoph J. Brabec, Miroslaw Batentschuk, Yuriy Zorenko

**Affiliations:** 1 Institute of Physics, Kazimierz Wielki University in Bydgoszcz, 85090 Bydgoszcz, Poland; gorbenko@ukw.edu.pl (A.M.); tetiana.zorenko@ukw.edu.pl (T.Z.); sanwit@ukw.edu.pl (S.W.-Ł.); 2 Institute of Materials for Electronics and Energy Technology (i-MEET), Department of Materials Science and Engineering VI, University of Erlangen-Nürnberg, 91058 Erlangen, Germany; yevgeniya.vlasyuk@fau.de (Y.V.); andres.osvet@fau.de (A.O.); jack.elia@fau.de (J.E.); christoph.brabec@fau.de (C.J.B.)

**Keywords:** WLED, phosphor converters, Ca^2+^-Mg^2+^-Si^4+^-based garnets, micropowder, luminescence, Ce^3+^ multicenters

## Abstract

This work is dedicated to the crystallization and luminescent properties of a prospective Ca_2_YMgScSi_3_O_12_:Ce (CYMSSG:Ce) micropowder (MP) phosphor converter (pc) for a white light–emitting LED (WLED). The set of MP samples was obtained by conventional solid-phase synthesis using different amounts of B_2_O_3_ flux in the 1–5 mole percentage range. The luminescent properties of the CYMSSG:Ce MPs were investigated at different Ce^3+^ concentrations in the 1–5 atomic percentage range. The formation of several Ce^3+^ multicenters in the CYMSSG:Ce MPs was detected in the emission and excitation spectra as well as the decay kinetics of the Ce^3+^ luminescence. The creation of the Ce^3+^ multicenters in CYMSSG:Ce garnet results from: (i) the substitution by the Ce^3+^ ions of the heterovalent Ca^2+^ and Y^3+^ cations in the dodecahedral position of the garnet host; (ii) the inhomogeneous local environment of the Ce^3+^ ions when the octahedral positions of the garnet are replaced by heterovalent Mg^2+^ and Sc^3+^ cations and the tetrahedral positions are replaced by Si^4+^ cations. The presence of Ce^3+^ multicenters significantly enhances the Ce^3+^ emission band in the red range in comparison with conventional YAG:Ce phosphor. Prototypes of the WLEDs were also created in this work by using CYMSSG:Ce MP films as phosphor converters. Furthermore, the dependence of the photoconversion properties on the layer thickness of the CYMSSG:Ce MP was studied as well. The changes in the MP layer thickness enable the tuning of the white light thons from cold white/daylight to neutral white. The obtained results are encouraging and can be useful for the development of a novel generation of pcs for WLEDs.

## 1. Introduction

With development of blue-emitting light-emitting diodes (LEDs) based on AlInGaN semiconductor chips, the lighting industry is in a technical breakthrough. In the past few years, these LEDs, in combination with blue-to-yellow light converters emitting white light (WLEDs), have caused quite a stir in the lighting industry. With their long service life, low production costs, and high efficiency, WLEDs are a suitable environmentally friendly alternative to conventional light bulbs and fluorescent lamps. They are also competitive thanks to their low energy consumption and resource-saving production. As a result, these diodes are significantly more ecological in terms of production and application than conventional light sources. They also have a useful color rendering index (CRI) and an easily adjustable correlated color temperature (CCT) [1,2,3,4,5]. LED technology can be found in the industry in many application areas, for example, in the automotive industry, in medical technology, and in lighting and sensor technology [6,7,8,9,10].

Development and spectroscopic investigation of Ca^2+^-Si^4+^-based garnets are very conducive for designing novel generations of phosphor converters (pc) in so-called *planar-type technology* for high-power WLED [11,12] and new materials for luminescent thermometry as well [13]. Nowadays, the development of such types of phosphors is a prospective direction in semiconductor lighting technology [12,13,14,15,16,17,18,19]. Currently, for the production of high-power WLEDs, YAG:Ce garnets in crystal or ceramic form are mainly used in combination with blue LED [11,20,21,22,23].

Of all the Ca-Si-based garnet phosphors, the most well-known is Ca_3_Sc_2_Si_3_O_12_ (CSSG) garnet, doped with different types of rare-earth or/and transition metal ions [11,20,24]. The simultaneous localization of Ce^3+^ ions and other rare-earth and transition metal ions in the different valence states in the dodecahedral sites enables the creation of a wide class of novel silicate-based garnet phosphors. Furthermore, additional Y^3+^-Mg^2+^ pair-alloying into the Ca_3_Sc_2_Si_3_O_12_:Ce garnet host opens additional possibilities for the modification of luminescent properties and the development of more efficient types of pc for WLED [13,25]. Namely, such an approach allows achieving the more suitable redshift of the emission spectra and better color properties of pc-WLEDs in comparison with conventional YAG:Ce pc [13,25].

The current work aims to study the characteristics of the luminescent properties of Ce^3+^-doped Ca_2_YMgScSi_3_O_12_ (CYMSSG:Ce) microcrystalline powder (MP) phosphors prepared using conventional solid-state synthesis, which can be used as an efficient pc for the creation of high-power WLEDs. Prototypes of pc-WLEDs based on CYMSSG:Ce MP planar layers of different thicknesses were fabricated in this work and their pc properties were investigated as well. The development of this type of phosphor is nowadays a hot topic in lighting technology, especially given that currently only phosphors based on YAG:Ce crystal or ceramic garnet are available for the production of power WLEDs with the excitation of blue LEDs.

## 2. Synthesis of CYMSSG:Ce Micropowder Samples and Their Structural Qualities

CYMSSG:Ce MP were synthesized by conventional solid synthesis as an effective production method based on a solid–solid reactions, and in this case, between the microcrystalline grains of the raw components. During the synthesis of MPs from CaO, Y_2_O_3_, MgO, Sc_2_O_3_, and SiO_2_ raw oxides with 4N purity, they were first weighed and then ground in agate solution for 20 min to obtain the greatest homogeneity of the powder. It is necessary to note here that the homogeneity of the samples significantly enhances the luminescent properties of the phosphors. The final phosphor mass was baked in an Al_2_O_3_ crucible at a heating rate of 20 °C/min to 1300 °C for 10 h in a reducing atmosphere (95% N_2_ +5% H_2_).

**Figure 1:**
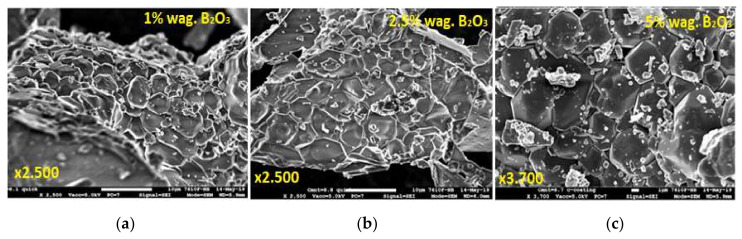
SEM images of CYMSSG: 2.5%Ce MPs, prepared using different amounts of B_2_O_3_ flux agent: (**a**) 1 wt.%, (**b**) 2.5 wt.%, and (**c**) 5 wt.%.

**Figure 2:**
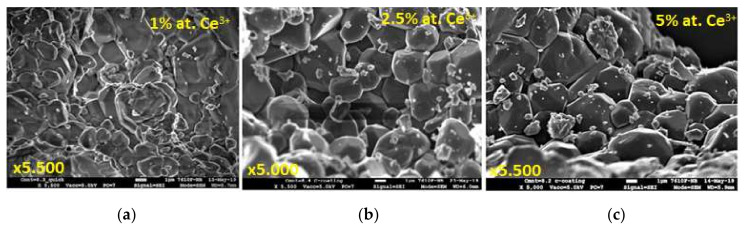
SEM images of CYMSSG:Ce MPs samples with different Ce^3+^ concentrations, prepared using 2.5 wt.% B_2_O_3_ flux agent: (**a**) 1 at.%, (**b**) 2.5 at.%, and (**c**) 5 at.%.

In the solid-state method, the preparation of phosphors using flux can substantially support the formation of the garnet phase and enables the obtaining of microparticles with high quantum yield. The application of flux can also significantly improve the morphology of the grains and, in this way, additionally enhance the material PL intensity. For this reason, the synthesis of CYMSSG:Ce MP samples was performed using B_2_O_3_ flux with concentration in the 1–5 wt.% range with respect to the total weight of the garnet charge. By using B_2_O_3_ flux to improve the condition of the solid-state reaction, the Ca_2_YMgScSi_3_O_12_ garnet phase in the MP samples under study was successfully obtained (Figure 1 and Figure 2).

Figure 1 demonstrates the SEM images of the CYMSSG:Ce MP samples synthesized from the charge containing 1 wt.%, 2.5 wt.%, and 5 wt.% B_2_O_3_ flux agent. Overall, the structure of the garnet is visible in all MP samples under study. From Figure 1, the influence of the flux and its concentration on the formation of the cubic particles with a garnet structure can also be observed. Cubic grains corresponding to the garnet structure are present in small amounts in the MP sample synthesized at a concentration of 1 wt.% B_2_O_3_ (Figure 1a). Furthermore, strong grain agglomeration is visible for this MP sample. On the contrary, the MP samples synthesized from compounds with a higher B_2_O_3_ content (Figure 1b,c) have a more homogeneous garnet structures, especially for the sample with the highest (5 wt.%) flux concentration (Figure 1c). That means that increasing the flux content leads to a uniformity of the grain distribution and some increase in the average grain size, up to 5–8 μm for a Ba_2_O_3_ concentration of 5 wt.%.

SEM images of CYMSSG:Ce MPs synthesized with an activator concentration in the 1–5% range are shown in Figure 2. Generally, garnet grains are visible in all samples. However, the strong grain agglomeration is observed for samples with the lowest Ce^3+^ content of 1%. Furthermore, the quantity of agglomerates is significantly lower in the samples with Ce^3+^ contents of 2.5 and 5 at.%, especially in the last one (Figure 2c). For the last sample, the most uniform distributions of the grain are observed, with their average size in the 3–5 μm range.

**Figure 3:**
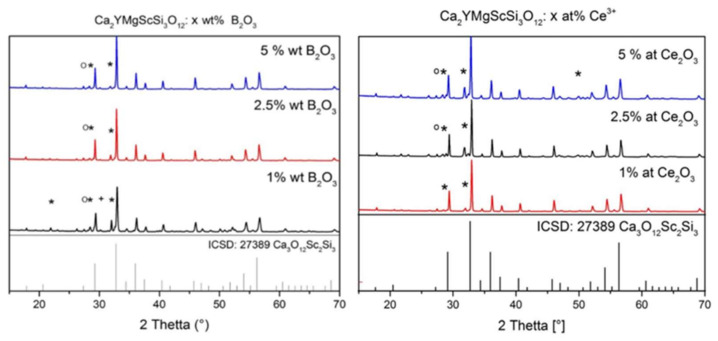
XRD patterns of CYMSSG:Ce MPs with different flux and activator amounts: (**a**) x wt.% B_2_O_3_ and (**b**) x at.% CeO_2_, where * is Ca_2_Ce_8_O_26_Si_6_, **+** is SiO_2_, and **o** is YBO_3_.

The X-ray diffraction (XRD) of MP samples sintered with different B_2_O_3_ flux amounts (Figure 3a) and different Ce^3+^ concentrations shows almost pure phases of Ca_3_Sc_2_Si_3_O_12_ garnet (CSSG) in both cases and excellent correspondence with the respective ICSD-27389 pattern. However, some minor amounts of unreacted starting materials, (SiO_2_) as well as the secondary phases (Ca_2_Ce_8_O_26_Si_6_, YBO_3_), are also observed in the XRD patterns (Figure 3).

From the XRD data, a garnet phase with maxima corresponding mostly to Ca_3_Sc_2_Si_3_O_12_ was detected. The main peak of the garnet at about 32.7° (2θ), which belongs to the {024} family of lattice planes, is very well defined in these compositions. However, additional peaks, which cannot be precisely identified, can be seen in Figure 3. 

It is noticeable here that the peaks are shifted by several 2θ. The incorporation of Mg^2+^ ions into the crystal lattice is a probable reason for the discernible shift to the right. The Mg^2+^ ions take the lattice sites of the Sc^3+^ ions. Due to the smaller ionic radius, the lattice spacing increases, which means that 2θ increases accordingly. As a result of the substitution with Y^3+^ and Mg^2+^, a newly modified silicate garnet structure that is not yet known in the database has probably emerged.

Furthermore, the increase in B_2_O_3_ achieves better single-phase character. This is illustrated by the peaks at around 32° (2θ) and between 51 and 52° (2θ) (Figure 3a). The proportion of the Ca_2_Ce_8_O_26_Si_6_ foreign phase and the intensity of the corresponding peak decreases with the increasing B_2_O_3_ concentration.

As expected, a garnet phase similar to that of Ca_3_Sc_2_Si_3_O_12_ could also be detected in a series of measurements with different concentrations of Ce ions using XRD analysis (Figure 3b). The main peaks of the silicate garnet phase are consistent with those of synthesized samples. However, as already mentioned, these are shifted. By increasing the CeO_2_ concentration, the intensity of the peak at 33° increases, and the proportion of the Ca_2_Ce_8_O_26_Si_6_ foreign phase increases as well.

## 3. Photoluminescence Quantum Yield of CYMSSG:Ce MPs

The photoluminescence quantum yield (PLQY) of the CYMSSG:Ce MPs depending on the synthesis conditions is given in Table 1. The PLOY values ranges lie between 42.1 and 63.6%, depending mainly on the content of the garnet phase in the MP samples under study. The highest quantum efficiency has the MP sample with an activator concentration of 2.5 at.% and a B_2_O_3_ flux agent content of 2.5 wt.%. Correspondingly, the garnet content in these MP samples is highest as well and equal to 82% (Table 1). It is necessary to note that second phases do not influence the emission properties in the Ce^3+^ emission spectral region and serve as light scattering centers and, probably, as emission centers in the UV region in the samples investigated in this work (see results below).

The largest garnet phase content, in the 80–82% range, is observed for the MP samples sintered with a Ba_2_O_3_ flux content in the 2.5–5 at.% range and the activator concentration in the 1–2.5% range. The most optimal condition is the sintering with a flux content of 2.5 wt.% B_2_O_3_ and a Ce^3+^ concentration of 2.5 at.%, enabling 82% garnet content in the MP sample (Table 1).

Some ways of improving the PLQY were investigated. For instance, an efficiency of more than 71% was achieved by the synthesis of CYMSSG:Ce in pellets by a two-step synthesis process. This was a case in which only 68% of the main phase was detected by XRD analysis.

## 4. Luminescent and Photoconversion Properties of CYMSSG:Ce MP Samples

For characterization of the optical properties of the CYMSSG:Ce MPs under study, the cathodoluminescence (CL), photoluminescence emission (PE) and excitation spectra (PLE), PL decay kinetics, and PLQY and photoconversion spectra (PC) measurements were used. The CL spectra were investigated using the e-beam from a scanning electron microscope SEM JEOL JSM-820 (JEOL, Warsaw, Poland) additionally equipped with a Stellar Net spectrometer with a TE-cooled CCD detector working in the 200–1200 nm range. PL and PLE spectra and PL decay kinetics were measured using an FS-5 spectrometer (Edinburgh Instruments, Livingston, UK). An EPL-450 picosecond pulsed diode laser was used to measure of the decay kinetics, and the typical average power of this laser is 0.15 mW (Edinburgh Instruments). The PC spectra and PLQY measurements were performed using a fiber-optic spectrophotometer AvaSpec-ULS 2048-LTEC (Avantes, Apeldoorn, The Netherlands), and an integrating sphere AvaSphere-50-IRRAD. The photoconverter (pc) prepared from CYMSSG:Ce MP films of different thicknesses was excited by the blue LED at a wavelength of λ = 450 nm. All luminescence measurements were performed at room temperature (RT).

### 4.1. Cathodoluminescence Spectra

**Figure 4:**
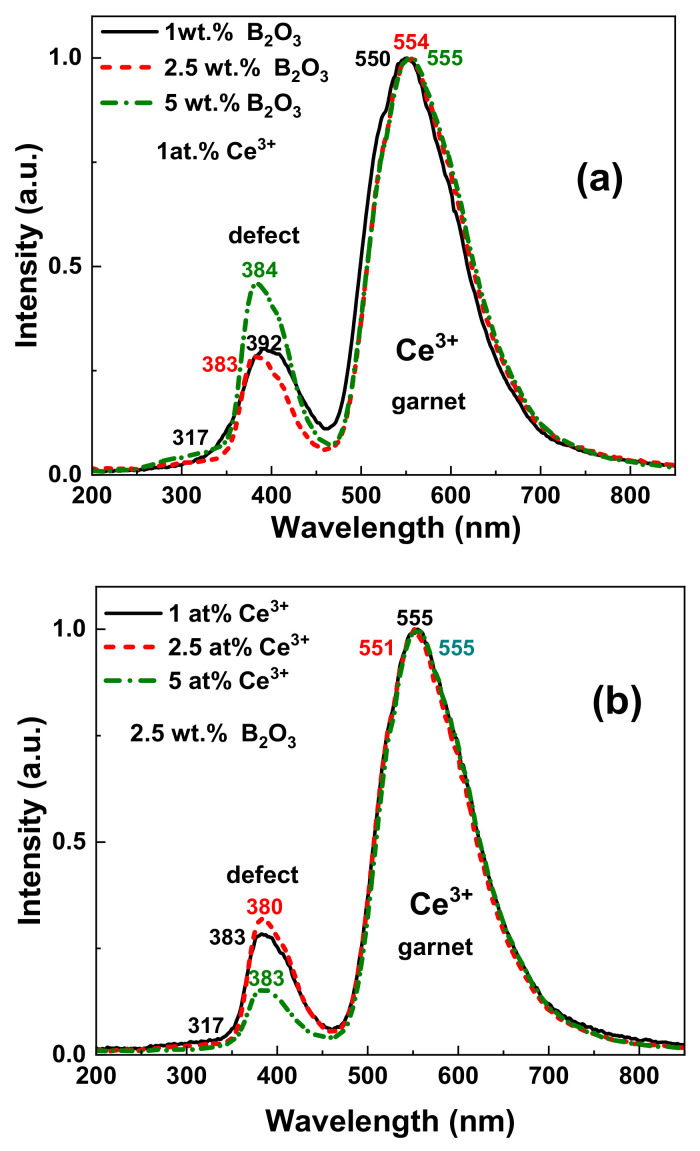
Normalized CL spectra of CYMSSG:Ce MPs sintered with different amounts of B_2_O_3_ flux (**a**) and different concentrations of Ce^3+^ ions (**b**).

The normalized CL spectra of the CYMSSG:Ce MPs samples sintered with different amounts of B_2_O_3_ flux and different concentrations of Ce^3+^ ions are shown in Figure 4a,b, respectively. The dominant luminescence band with a peak in the 550–555 nm range in all MP CYMSSG:Ce samples corresponds to the 5d^1^ → 4f (^2^F_5/2,7/2_) transitions of Ce^3+^ in these garnet compounds. The position of the Ce^3+^ band is slightly red-shifted from 551 to 555 nm in the MP samples with an increase of the flux contents from 1 wt.% to 5 wt.% (Figure 4a) and Ce^3+^ content from 1 to 5% (Figure 4b).

Apart from the luminescence of the Ce^3+^ ions in the garnet structure in the visible range, the other emission bands in the UV range peaked in the 380–392 nm range are also observed in the CL spectra for the CYMSSG:Ce MPs. Furthermore, the lowest UV luminescence efficiency is observed in the CL spectra of the CYMSSG:Ce MP sample sintered with flux and activator contents of 2.5 wt.% and 5 at.%, respectively (Figure 4b).

It is important to note here that the low intensity bands in the UV range of Ca^2+^-Mg^2+^-Si^4+^ -based garnets usually are assigned to defect luminescence [26,27]. According to the authors [26,27], the presence of a high concentration of oxygen vacancies in these gannets is expected due to possible deviations in the concentration of heterovalent Ca^2+^, Mg^2+^, and Si^4+^ cations and the demands for local charge compensation. For this reason, the bands peaked at 317 nm and the 380–392 nm range may correspond to the luminescence of the F^+^ and F centers (single- and double-charged oxygen vacancies, respectively) in the CYMSSG host [27,28]. However, it is also worth mentioning here that, due to the relatively large contents of the secondary phases in the tested MP samples, the luminescence spectra in the UV range may also partly correspond to the Ce^3+^ luminescence in these compounds. However, a visible correlation between the contents of the secondary phases and the intensity of the UV luminescence was not observed for the tested CYMSSG:Ce MP samples.

### 4.2. PL and PLE Spectra

**Figure 5:**
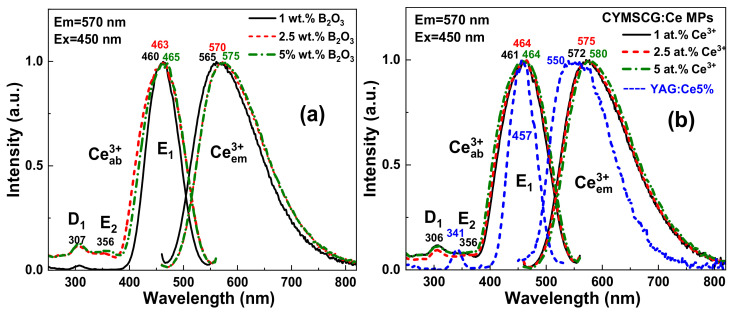
PL spectra and PLE spectra of CYMSSG:Ce (1 at.%) MPs sintered with different amounts of B_2_O_3_ flux (**a**) and PL spectra and PLE spectra of CYMSSG:Ce MPs samples with different concentrations of Ce^3+^ ions sintered with a B_2_O_3_ flux content of 2.5 wt.% (**b**).

The PL spectra of the CYMSSG:Ce (1 at.%) MPs (Figure 5a) sintered with different B_2_O_3_ flux amounts show wide bands peaked in the 565–575 nm range, which corresponds to the Ce^3+^ radiation transitions from the lowest 5d^1^ level of the excited state to the 4f(^2^F_5/2,7/2_) levels of the ground state. Increasing the concentration of B_2_O_3_ leads to a shift in the emission spectra of the Ce^3+^ ions to the long-wavelength spectral range (Figure 5a) [11,27,28,29].

The PLE spectra of these MP samples (Figure 5b) show several bands in the 250–550 nm region. The main band E_1_ peaked in the 460–465 nm range is explained by the absorption of the allowed transitions from the 4f ground state to the lowest 5d^1^ levels of the Ce^3+^ ions. Moreover, two MP samples, synthesized with a higher concentration of boron oxide, have a E_2_ band peaked at 356 nm, which also corresponds to the 4f (5d^2^) excitation band of the Ce^3+^ ions. The low intensity of the E_2_ band in comparison with conventional YAG:Ce garnets (Figure 4b) is characteristic of Ca^2+^ (or Mg^2+^) -Si^4+^-based garnets (see also [27,30,31]).

Figure 5b demonstrates the PL and PLE spectra of the CYMSSG:Ce MPs with variable Ce^3+^ ion concentrations in the range 1–5 at.% synthesized with a flux B_2_O_3_ concentration of 2.5 wt.%. Increasing the Ce^3+^ concentration in these MP samples leads to the increases of the crystal field strength in the dodecahedron position of the garnet compounds, which results in a long-wavelength shift in the Ce^3+^ emission spectrum (Figure 5b). Moreover, the PL emission and excitation spectra of all CYMSSG:Ce MP samples is considerably broadened in comparison with the conventional CSSG:Ce and YAG:Ce phosphors (Figure 5b). Namely, the PL emission spectrum in CYMSSG:Ce MPs shifted with respect to YAG:Ce spectrum by more than 40 nm into the red range, extending up to even 800 nm. The respective FWHM of the Ce^3+^ emission bands increases from 124.5 nm in the YAG:Ce 5% to 139.5 nm in the CYMSSG:Ce5% MPs. The FWHM of the main Ce^3+^ excitation band in the PLE excitation spectrum of the CYMSSG:Ce MPs is also significantly larger in the CYMSSG:Ce5% MPs (104 nm) in comparison with the YAG:Ce5% sample (56 nm) (Figure 5b). Such results, without any doubt, indicate the existence of different Ce^3+^ centers in the CYMSSG garnets (see also [27,30,31,32,33] for details).

It is also worth noting that the PLE spectra of these MP samples (Figure 5a) also contain a band in the UV range peaked at 308 nm, which can correspond to the excitation of the defect luminescence in the CYMSSG host in the bands peaked in the 380–393 nm range (Figure 2). Such emission bands are overlapped with the E_1_ and E_2_ excitation bands of the Ce^3+^ luminescence in the garnets. That results in the excitation of the Ce^3+^ luminescence via the luminescence of the defects centers. Thus, energy transfer between the defect and Ce^3+^ centers is observed in all CYMSSG:Ce MP samples under study.

**Figure 6:**
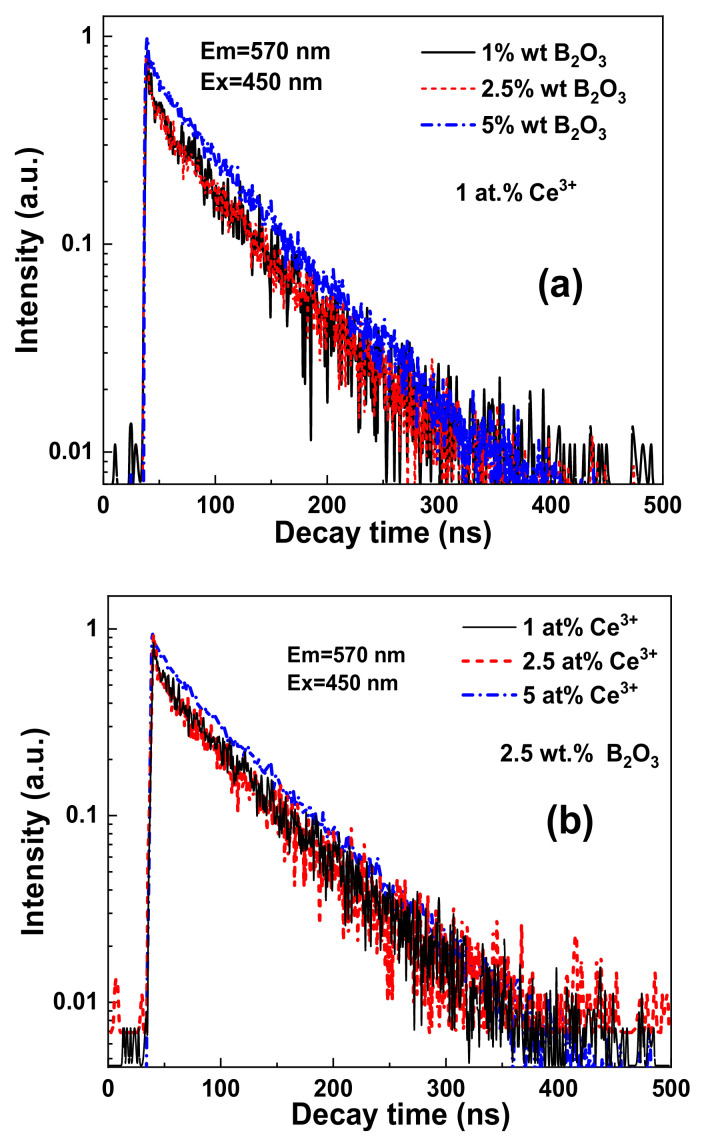
PL decay kinetics at RT recorded at 570 nm under blue light excitation (450 nm) in MPs sintered with different amounts of B_2_O_3_ flux (**a**) and different concentrations of Ce^3+^ ions (**b**).

Figure 6 shows the PL decay kinetics of the MP CSSG: Ce samples with different concentrations of B_2_O_3_ flux (a) and Ce^3+^ activator (b). The approximation parameters of the respective decay curves are given in Table 2 and Table 3, respectively.

Similarly to other Ca^2+^-Si^4+^-based garnets [26,32,33], the decay kinetics of the Ce^3+^ emission in Ca_2_YMgScSi_3_O_12_:Ce MPs is non-exponential. For this reason, the three-exponential fit of the decay curves was used for the quantitative description of the luminescence timing properties (Table 1 and Table 2). Although such a three-exponential approximation does not describe the luminescence decay behavior correctly at quenching due to the energy transfer, the decay time values can be considered as estimations of the luminescence decay times of the respective Ce^3+^ multicenters.

## 5. Ce^3+^ Multicenter Formation in CYMSSG:Ce Phosphor

**Figure 7:**
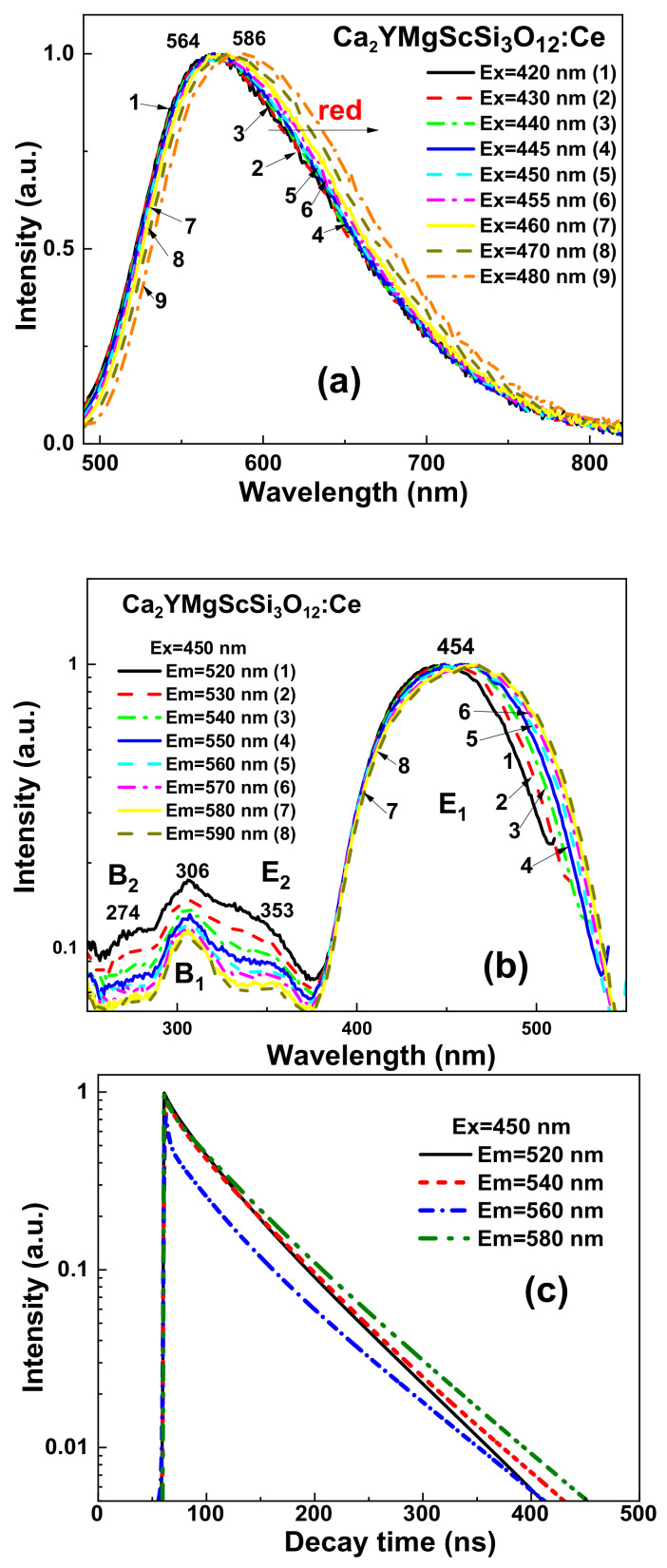
Detailed luminescent properties of Ca_2_YMgScSi_3_O_12_:Ce5% MPs at RT. Emission (**a**) and excitation (**b**) recorded in different parts of the respective spectra. PL decay kinetics (**c**) at RT recorded under blue light (450 nm) excitation in different parts of the Ce^3+^ emission band.

**Figure 8:**
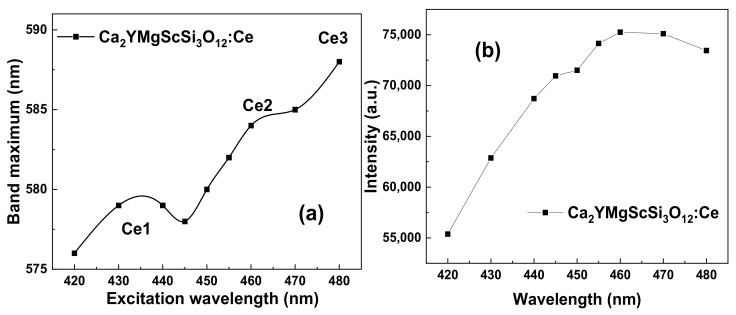
Dependencies of the maxima (**a**) and intensities (**b**) in the peak positions of Ce^3+^ emission bands on excitation wavelength in Ca_2_YMgScSi_3_O_12_:Ce MPs (see Figure 7a,b).

The RT PL emission spectra of the CYMSSG:5%Ce MPs are shown in Figure 7a under excitation in the characteristic PLE bands (Figure 7b). The PL spectra of these samples show the intensive luminescence in the form of wide bands peaked in the green range related to the 5d^1^→4f(^2^F_5/2,7/2_) transitions of the Ce^3+^ ions. Moreover, by increasing the excitation wavelength from 420 to 480 nm, the PL spectra of the CYMSSG:Ce MPs are significantly red-shifted from 564 to 587 nm and slightly narrowed (Figure 7a). (Figure 7a, curves 1–9, respectively). Furthermore, the shift in the main maxima of the PL spectra of the CYMSSG:Ce MPs and the intensity in the peak positions occurs non-monotonically with the increasing excitation wavelength (Figure 8a,b) in comparison with the similar dependencies for the YAG:Ce SCF sample (not shown in Figure 8). Behavior such as that shown in the PL emission spectra indicates the Ce^3+^ multicenter formation in the CYMSSG:Ce garnet.

The normalized excitation spectra of the Ce^3+^ luminescence in the Ca_2_YMgScSi_3_O_12_:Ce MP samples are shown in Figure 7b. In these spectra, the most intensive excitation bands, E_1_ and E_2_, peaked in the 454 nm (E_1_) and 353 nm (E_2_) ranges, are attributed to the intrinsic transitions from the 4f(^2^F_5/2_) level of the ground state to the 5d (E_2_) excited level of the Ce^3+^ ions (Table 4). The excitation bands peaked at 306 nm (B_1_ band) and 274 nm (B_2_ band) are also observed in the excitation spectra of the Ca_2_YMgScSi_3_O_12_:Ce MPs (Figure 7a,b). These excitation bands are not related to the intrinsic transitions of the Ce^3+^ ions and probably correspond to the Ce^3+^ luminescence excitation by the emission of the defect centers or the emission of flux-related impurities [30,31].

The decay kinetics of the Ce^3+^ luminescence in the Ca_2_YMgScSi_3_O_12_:Ce MPs registered at 520–580 nm under excitation at 450 nm are shown in Figure 7c. Due to the strong non-exponentiality of the decay kinetics, the three-exponential fit of the decay curves was used for the quantitative description of the luminescence timing properties (Table 5).

**Figure 9:**
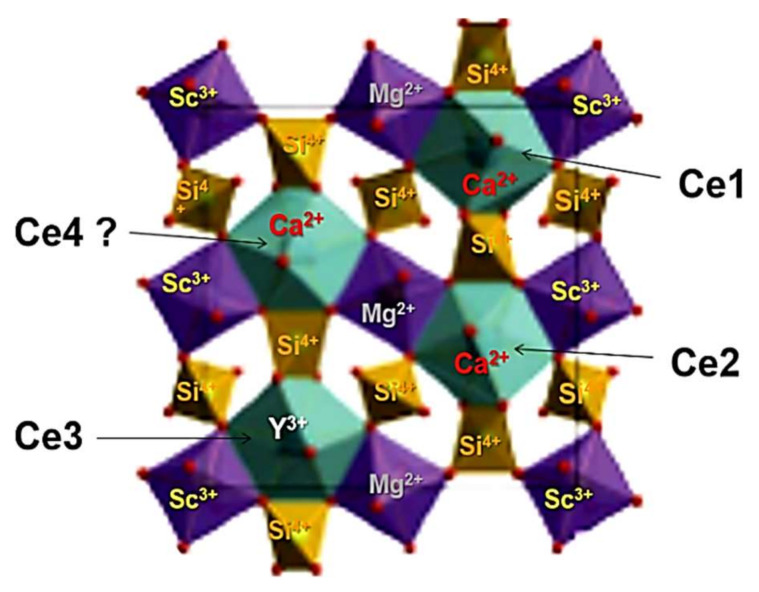
Visualization of possible Ce^3+^ multicenter formation in the structure of Ca_2_YMgScSi_3_O_12_:Ce garnet.

Behavior such as that shown in the decay curves of the Ca_2_YMgScSi_3_O_12_:Ce MPs can also indicate possible energy transfer from high-energy to low-energy Ce^3+^ emitting centers in these garnets [32,33]. The different decay times in the Ca_3_Sc_2_Si_3_O_12_:Ce SCF can correspond to the different Ce^3+^ centers in the dodecahedral positions of the garnet lattice with various local surroundings by oxygen and cations (Sc^3+^ and Si^4+^ ions in the octahedral and tetrahedral positions, respectively, and Ca^2+^ and Y^3+^ cations in the dodecahedral positions of the garnet host; Figure 9).

## 6. WLED Prototype Creation

**Figure 10:**
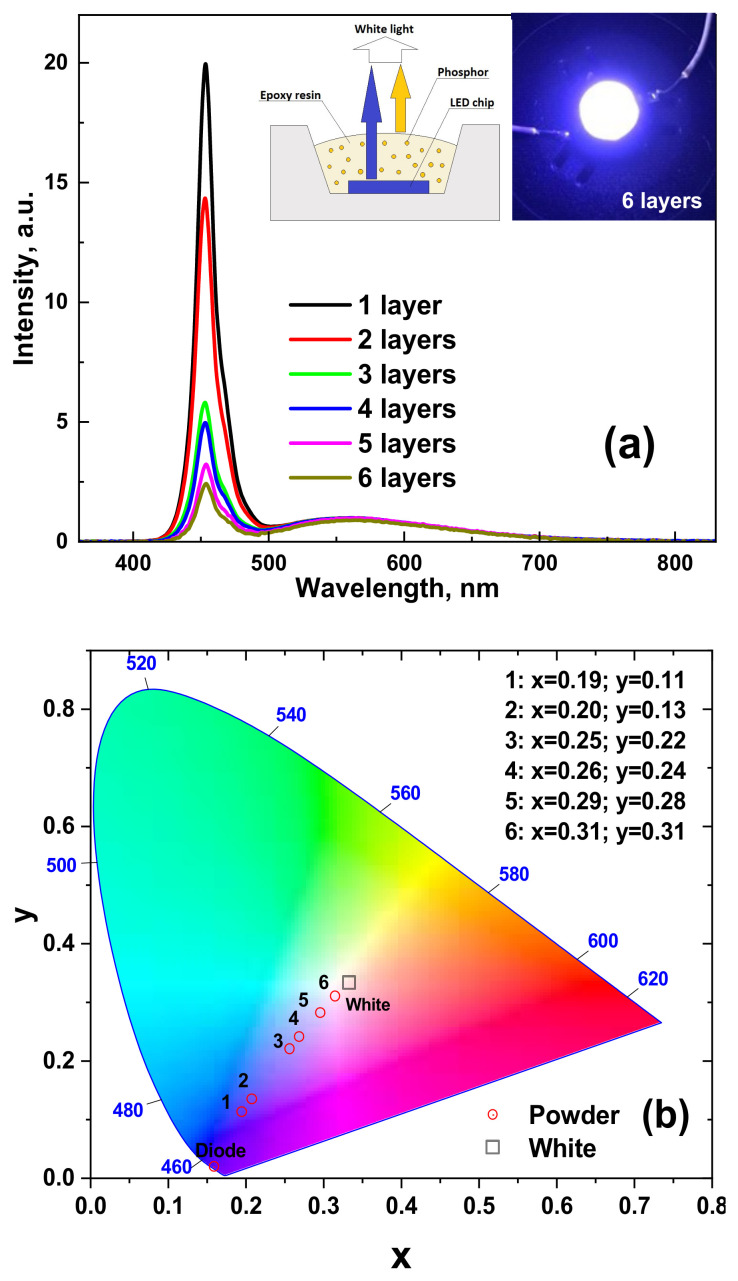
Emission spectrum (**a**) and chromaticity diagram (**b**) of a WLED lamp fabricated on the base of 450 nm LED chip and CYMSSG:1%Ce phosphor.

To demonstrate the application possibility of the development of CYMSSG:Ce phosphor, WLED prototypes were fabricated by coating a blue LED with an emission wavelength of 450 nm with several films containing CYMSSG:Ce phosphor embedded in epoxy resin (Figure 10a). PC measurements of the WLED prototypes were performed after each successive PC layer of a thickness of approximately 100–120 µm. The emission spectra of these WLEDs are quite broad and cover the entire visible range from 400 to 780 nm. The emission spectra of the WLED prototypes also show a significant decrease in the intensity of the blue component and a respective continuous increase in the intensity of the yellow emission by increasing the total film thickness (Figure 10a). As can be seen from Figure 10b, the color coordinates move to the diagram’s center by increasing the total thickness of the PC film. Finally, the WLED prototypes, based on six films of CYMSSG:Ce MP PC, give quite cold white emissions with the correlated color coordinates (CCC) x = 0.315; y = 0.31 and a correlated color temperature (CCT) of 6930 K. Furthermore, as the thickness of the photoconverter film increases, the color coordinates move to the center of the diagram, including the warm white color pattern. However, these trends of the photoconverting properties of the developed WLED prototypes are quite limited by the conversion efficiency of the CYMSSG:Ce MP phosphor with a Ce^3+^ concentration of 1 at.%. To obtain a precise tuning of the photoconversion properties (CCC and CCT), the CYMSSG:Ce MP phosphor with Ce^3+^ concentration above 1% can be used as well.

The fabricated device with bright-light characteristics demonstrates that the WLEDs based on the CYMSSG:Ce MPs are very promising and can be applied to lighting systems (Figure 10a, insert). The CIE chromaticity coordinates of the prototype WLEDs are shown in Table 6.

## 7. Conclusions

Microcrystalline powder (MP) samples of Ca_2_YMgScSi_3_O_12_:Ce garnet with Ce^3+^ concentrations in the range of 1–5 at.% were obtained using solid-state synthesis by adding B_2_O_3_ flux in the 1–5 wt. % concentration of the total charge content. To determine the luminescent properties of the CYMSSG:Ce MPs, the cathodoluminescence (CL) spectra, photoluminescence (PL) emission and excitation spectra, PL decay kinetics, and the PL quantum yields (PL QY) were measured.

The obtained results confirm the Ce^3+^ multicenter formation in the MP of the CYMSSG:Ce garnet. It is mainly caused by the location of the Ce^3+^ ions in the dodecahedral positions of the Ca^2+^ and Y^3+^ cations, and the different local surroundings of these centers. Namely, Ce^3+^ multicenters such as those in the mentioned dodecahedral positions of the garnet host possess additional local asymmetry and crystal field strength due to inhomogeneity in the local environment of these positions during the substitutions at the octahedral sites by heterovalent Mg^2+^ and Sc^3+^ ions and the at the tetrahedral sites by Si^4+^ ions. Based on the results of the optical investigations, the luminescent characteristics of different Ce^3+^-based multicenters were estimated.

The application potential of the developed CYMSSG:Ce MP phosphor was demonstrated. Planar WLED prototypes were fabricated by coating GaN 450 nm blue LED chips with several 100–120 μm thick layers of CYMSSG:Ce MP phosphor mixed with epoxy resin. Furthermore, the WLED prototypes, based on five CYMSSG:Ce MP photoconverters, give emissions close to white light with the coordinates x = 0.315; y = 0.31 and a color temperature of 6930 K.

## Figures and Tables

**Table 1:** The garnet/secondary phase proportions and PLQY of the CYMSSG:Ce MPs sintered with different flux and activator contents.

Nominal Chemical Composition Ca_2_MgYScSi_3_O_12_:Ce	Garnet Content, %	Secondary Phases Content, (%)	PLQY, %
1 at.% Ce^3+^ + 1 wt.% B_2_O_3_	49.5	CaO (10.9); SiO_2_ (21.8) YBO_3_ (5); Ce_2_O_3_ (12.9)	42.1
1 at.% Ce^3+^ + 2.5 wt.% B_2_O_3_	80	Ca_2_Ce_8_O_26_Si_6_ (11); SiO_2_ (7); CaO_2_ (2)	54.5
1 at.% Ce^3+^ + 5 wt.% B_2_O_3_	80	Ca_2_Ce_8_O_26_Si_6_ (9) SiO_2_(4); Ce_2_O_3_ (5); YBO_3_ (2)	47.9
1 at.% Ce^3+^ + 2.5 wt.% B_2_O_3_	81	Ca_2_Ce_8_O_26_Si_6_ (9); SiO_2_ (1); Ce_2_O_3_ (2); MgO (2); Ca (5)	48.5
**2.5 at.% Ce^3+^+ 2.5 wt.% B_2_O_3_**	**82**	**Ca_2_Ce_8_Si_6_O_26_ (14); Ce_2_O_3_ (2); SiO_2_ (2);**	**63.6**
5 at.% Ce^3+^ + 2.5 wt.% B_2_O_3_	62	Ca_2_Ce_8_O_26_Si_6_ (13); SiO_2_ (17) CaO_2_ (2); MgO (6)	44.3

**Table 2:** Parameters of three exponential approximations of the decay curves presented in Figure 6a (Em = 570 nm, Ex = 450 nm).

x% wag. B_2_O_3_	t_1_, ns	A_1_	t_2_, ns	A_2_	t_3_, ns	A_3_
1	4.44	21.59	42.1	21.58	66.88	21.78
2.5	5.31	8.12	54.5	29.46	68.17	29.75
5	3.93	53.17	47.9	48.64	58.62	55.74

**Table 3:** Parameters of three exponential approximations of the decay curves presented in Figure 6b (Em = 570 nm, Ex = 450 nm).

x% at. Ce^3+^	t_1_, ns	A_1_	t_2_, ns	A_2_	t_3_, ns	A_3_
1	8.49	26.79	51.65	65.93	80.18	57.11
2.5	6.90	26.87	72.03	34.59	73.13	32.18
5	11.54	29.05	72.79	33.99	79.74	42.03

**Table 4:** Spectral characteristics of the different Ce^3+^ multicenters in Ca_2_YMgScSi_3_O_12_:Ce garnet.

Type of Centers	Maximum of Dominant Emission Band, nm	Position of E_2_ and E_1_ Excitation Bands, nm	ΔE = E_2_ − E_1_, eV	Stokes Shift, eV
Ce1	569	349;446	0.773	0.601
Ce2	573	358;458	0.756	0.542
Ce3	586	354;461	0.813	0.574

**Table 5:** Parameters of three exponential approximations of the decay curves presented in Figure 7c.

Emission Wavelength	t_1_, ns	A_1_	t_2_, ns	A_2_	t_3_, ns	A_3_
520 nm	6.38	0.05	36.32	0.39	77.64	0.48
540 nm	15.47	0.12	66.76	0.61	79.04	0.51
560 nm	2.54	0.85	37.34	0.29	90.71	0.25
580 nm	5.31	0.14	44.72	0.34	85.19	0.49

**Table 6:** CIE chromaticity coordinates of a WLED lamp fabricated on the base of 450 nm LED chip and CYMSSG:1%Ce phosphor.

Samples	Unpolished Samples (h = 1 mm)	
	CIE Coordinates	
	x	y
1 layer	0.195	0.112
2 layers	0.208	0.134
3 layers	0.257	0.220
4 layers	0.279	0.241
5 layers	0.296	0.282
6 layers	0.315	0.31

## Data Availability

Not applicable.
